# Differential Rates of Lower Gastrointestinal Bleeding and Other Outcomes in Colorectal Cancer Patients With Aortic Stenosis

**DOI:** 10.7759/cureus.35926

**Published:** 2023-03-09

**Authors:** Ayham Khrais, Nimra Gilani, Jared Sapin, Yazan Abboud, Aaron Kahlam, Alexander Le, Meet Shah, Arthi Palani, Jahanzeb Javed

**Affiliations:** 1 Internal Medicine, Rutgers University New Jersey Medical School, Newark, USA; 2 Hospital-Based Medicine, Rutgers University New Jersey Medical School, Newark, USA

**Keywords:** iron deficiency anemia (ida), acute gi bleed, heyde syndrome, colorectal cancer, aortic stenosis (as)

## Abstract

Background

Aortic stenosis (AS) has been established as a precipitating factor in the development of colonic angiodysplasia, resulting in lower gastrointestinal bleeding (LGIB). While the association between AS and LGIB, termed “Heyde syndrome,” has been examined extensively, few studies assess the impact of comorbid AS on rates of LGIB in patients with colorectal cancer (CRC). Our goal is to examine this association.

Methods

Patients hospitalized from 2001 to 2013 diagnosed with CRC were identified via ICD-9 codes, further stratified by a diagnosis of AS. Continuous and categorical variables were analyzed by independent sample t-tests and chi-squared analyses respectively. Assessed outcomes included mortality, length of stay (LOS), hospital costs, rates of LGIB, colonic obstruction, colonic perforation, iron-deficiency anemia (IDA), and colectomy. Multivariate analysis via binary logistic regression was utilized to control confounding variables.

Results

Patients with CRC and AS had higher rates of mortality, lower gastrointestinal bleeding, iron deficiency anemia, and colectomy, while those without AS had higher rates of colonic obstruction. Length of stay and total hospital charges were higher in patients with AS.

Discussion

CRC outcomes were worse in patients with AS. This could be due to higher rates of LGIB secondary to the prevalence of angiodysplasia among AS patients. More retrospective studies are required to assess the impact of comorbid AS in patients with CRC.

## Introduction

Colorectal cancer (CRC) is the third-most common malignancy and the second-most common cause of cancer-related death in the US [1]. The incidence of CRC within the US has been decreasing, now down to approximately 140,000 new cases yearly [1,2]. The disease most commonly affects the proximal colon (which is more prone to bleeding), followed by the distal colon and then the rectum [2]. Patients often present with iron deficiency anemia (IDA), bright red blood per rectum (BRBPR), weight loss, and changes in bowel habits [2]. IDA has been shown to be a poor prognostic indicator in patients with CRC grade 3 [3]. IDA and lower gastrointestinal bleeding (LGIB) in CRC patients can be further compounded by coexisting factors, including the use of antiplatelet agents, anticoagulation, and inherent defects in platelet aggregation and the clotting cascade. Acquired von Willebrand syndrome (AvWS) is one such disorder, in which a decrease in circulating levels of von Willebrand factor (vWF) exists due to either anti-vWF antibodies or disruption of vWF multimers secondary to proteolysis [4]. Fragmentation of vWF multimers can result from uremia, cirrhosis, or from aortic stenosis (AS) [4]. In AS, excess shearing of vWF, caused by high velocities during passage through the stenosed aortic valve (AV), results in both mechanical damage and activation of ADAMTS-13 metalloproteinase [4-6]. Consequently, due to the lack of vWF-mediated platelet adhesion, patients exhibit prolonged bleeding time, predisposing them to increased rates of bleeding [4,6]. 

Heyde’s syndrome (HS) denotes the formation of bleeding colonic angiodysplasias due to AvWS resulting from AS [6]. HS is most common in patients 65 years of age or older, as are AS and the development of arteriovenous malformations (AVMs) [6,7]. Colonic AVMs are described as abnormal groups of dilated, tortuous blood vessels found within the colonic mucosa and submucosa [8]. Formation of these AVMs is thought to be secondary to compression of submucosal veins by the muscularis propria, resulting in venous backflow to the capillaries, and formation of alternate arteriovenous blood vessels [8]. These blood vessels have thin endothelial layers and are therefore more predisposed to damage and bleeding [6-8]. Colonic AVMs found in Western patients are most often right-sided, while those in Eastern patients are usually left-sided [8]. In effect, patients suffer a combination of symptoms resulting from these different disease processes, including LGIB, dyspnea, and potentially syncope. Management involves the repair of the stenotic AV, which would prevent further shearing of vWF multimers, thereby preventing the development and propagation of colonic AVMs [6]. 

Despite the pervasiveness of LGIB in patients with HS and CRC, the literature is scarce in assessing potential correlations among both disease processes, in spite of their many shared characteristics. Both HS and CRC increase in prevalence with age. Both can result in LGIB and IDA. Furthermore, the preponderance of right-sided AVMs (in Western HS patients) is likely to propagate any potential hemorrhage from bleeding colorectal tumors, which also usually develop from ascending colonic mucosa. We aim to study the association between these two disease processes. However, as HS is still underrecognized and underdiagnosed, we chose to study the correlation between AS and CRC instead. Specifically, our goal is to assess whether patients with AS, who would potentially develop AvWS, have differential rates of CRC complications (including LGIB and IDA) as opposed to those without AS.

## Materials and methods

Data source

The Nationwide Inpatient Sample (NIS) was used to analyze a sample of patients hospitalized from 2001 to 2013. International Classification of Diseases, 9th revision (ICD-9) codes were used to identify patients 18 years or older with a primary diagnosis of CRC, further stratified by the concomitant presence of AS (Table 1).

Statistical analysis

SPSS Statistics v. 24 (IBM Corp., Armonk, NY, USA) software was used to obtain and analyze data from the NIS. Continuous and categorical variables were analyzed by independent sample t-tests and chi-squared analyses respectively. Patient baseline characteristics, including age, sex at birth, and race were examined. Assessed outcomes included mortality, length of stay (LOS), hospital costs, rates of LGIB, colonic obstruction, colonic perforation, IDA, and colectomy. Multivariate analysis via binary logistic regression was utilized to control for confounding variables, including age, sex at birth, race, peptic ulcer disease, long-term use of anticoagulants, long-term use of antiplatelet agents, presence of mechanical valve prosthesis, atrial fibrillation, atrial flutter, von Willebrand disease, and hemophilia (including defects in Factors VIII, IX, and XI).

**Table 1:** Diagnoses and Associated ICD-9 Codes. ICD-9 = International Classification of Diseases, 9th Revision.

Diagnosis	ICD-9 Code
Colorectal Cancer	153.x
Aortic Stenosis	395.0, 396.2, 424.1, 746.3
Lower Gastrointestinal Bleeding	578.1
Intestinal Obstruction	560.9
Colonic Perforation	569.83
Iron Deficiency Anemia	280.0, 280.9
Colectomy	45.7x, 45.81-45.83
Long-Term Use of Anticoagulants	V58.61
Long-Term Use of Antiplatelets/Antithrombotics	V58.63
Mechanical Valve Prosthesis	V43.3
Atrial Fibrillation	427.31
Atrial Flutter	427.32
Von Willebrand Disease	286.4
Hemophilia	286.0-286.3

## Results

Among patients diagnosed with CRC from 2001 to 2013, 7,761 had concomitant aortic stenosis while 473,188 had no such diagnosis. Patients in both AS and non-AS groups were mostly female and Caucasian. The non-AS group had greater percentages of Black (13.3%) and Hispanic (7.4%) patients (Table 2).

**Table 2:** Demographics. n = sample size, SD = standard deviation.

		Patients without AS (n=473,188)	Patients with AS (n=7,761)
Age (SD)		68.68 years (13.7)	80.48 (9.1)
Sex	Male (%)	229,123 (48.4)	3,626 (46.7)
	Female (%)	243,842 (51.6)	4,135 (53.3)
Race	Caucasian (%)	280,073 (73.7)	5,400 (84.9)
	Black (%)	50,665 (13.3)	408 (6.4)
	Hispanic (%)	28,207 (7.4)	278 (4.4)
	Asian/Pacific Islander (%)	10,416 (2.7)	113 (1.8)
	Native American (%)	1,528 (0.4)	19 (0.3)
	Other (%)	9,282 (2.4)	141 (2.2)

In terms of outcomes, patients with AS had higher rates of mortality (6.2% versus 5.6%), LGIB (3.2% versus 1.8%), iron deficiency anemia (21.7% versus 11.5%), and colectomy (0.4% versus 0.2%). Patients without AS had higher rates of colonic obstruction (3.7% versus 2.6%). After adjustment for confounders, rates of mortality were statistically insignificant among both groups. Rates of colonic perforation were not significantly different among both groups (Table 3).

**Table 3:** Analysis of Categorical Variables. n = sample size, LGIB = lower gastrointestinal bleeding, IDA = iron deficiency anemia, CI = confidence interval, AOR = adjusted odds ratio, ACI = adjusted confidence interval.

	Patients without AS (n=473,188)	Patients with AS (n=7,761)	Odds Ratio (CI)	p-value	AOR (ACI)	Adjusted p-value
Mortality (%)	26,302 (5.6)	480 (6.2)	1.12 (1.02-1.229)	<0.05	0.986 (0.898-1.084)	0.774
LGIB (%)	8,284 (1.8)	247 (3.2)	1.845 (1.622-2.098)	<0.005	1.641 (1.442-1.868)	<0.005
Colonic Obstruction (%)	17,652 (3.7)	198 (2.6)	0.676 (0.586-0.779)	<0.005	0.703 (0.61-0.81)	<0.005
Perforation (%)	7,215 (1.5)	107 (1.4)	0.903 (0.745-1.094)	0.297	0.86 (0.71-1.04)	0.125
IDA (%)	54,308 (11.5)	1,687 (21.7)	2.142 (2.028-2.263)	<0.005	2.023 (1.915-2.138)	<0.005
Colectomy (%)	1,062 (0.2)	30 (0.4)	1.725 (1.199-2.481)	<0.005	1.632 (1.132-2.352)	<0.05

Patients with AS had prolonged LOS (9.2 days versus 7.76 days) and significantly more costly total hospital charges ($57,503.74 versus $45,289.36) with statistical significance (Table 4).

**Table 4:** Analysis of Continuous Variables. n = sample size, ACI = adjusted confidence interval, LOS = length of stay, USD = United States dollars.

	Patients without AS (n=473,188)			Patients with AS (n=7,761)			ACI	Adjusted p-value
	Mean	SD	SE Mean	Mean	SD	SE Mean		
LOS (days)	7.76	7.65	0.011	9.2	7.47	0.085	7.76 to 7.8	<0.005
Total Charges (USD)	$45,289.36	61,529.53	90.43	$57,503.74	67,405.76	773.4	45,310.38 to 45,662.65	<0.005

## Discussion

We found that CRC patients with AS have higher rates of LGIB and resulting IDA. Furthermore, mortality and colectomy rates were slightly more elevated in the AS group compared to their counterparts. We hypothesize that increased rates of bleeding in AS patients are likely due to AS-associated AvWS. Shearing from AS results in the cleavage of vWF multimers into smaller oligosaccharides, affecting its function in platelet aggregation while maintaining normal concentrations of vWF [9]. Dysfunction in vWF would subsequently predispose affected patients to increased bleeding rates [5,6,9]. Previous research has shown that AS severity is inversely related to concentrations of high molecular weight (HMW) vWF multimers, which is the larger vWF oligosaccharide subset [10]. Furthermore, elevated mean aortic gradients were associated with lower levels of HMW vWF [10]. As such, patients with high shear stress resulting from AV calcification are more predisposed to bleeding. Colonic tumors have superficial, thin-walled, fragile blood vessels [11]. As such, CRC patients already have a higher likelihood of LGIB than an individual without CRC. Therefore, if CRC patients also have an inherent defect in platelet aggregation (as is established in AS-induced AvWF), then rates of LGIB and resultant IDA are likely to be elevated.

First-line treatment of CRC, whether in its primary or metastatic stage, consists of resection when possible. Endoscopic removal of lesions is adequate in earlier cancers, however, certain high-risk features warrant definitive surgical treatment. For patients with a diagnosis of hereditary non-polyposis colon cancer (HNPCC), a total colectomy is recommended [12]. In our search, we found that CRC patients with AS had higher rates of colectomy. In the setting of concomitant aortic stenosis, surgical resection of the tumor carries an increased operative risk depending on the severity of the aortic stenosis, resulting in an increase in both mortality and major adverse cardiovascular events [13,14]. Pre-operative anemia specifically in the setting of colectomy is associated with an increased amount of 30-day post-operative complications as well as hospital re-admission rates [14]. This is complicated in part by the finding that transfusions are not benign in these patients and have been found to impair the immune response against the malignancy itself, and therefore lead to worsened outcomes. The recommendations in case of anemia in this population consist of erythropoietin and iron supplementation rather than transfusion [14]. Therefore it is not only the primary disease of CRC, but the indicated treatments as well that can lead to worsening outcomes in the setting of AS.

In our analysis, we found that CRC patients with AS had longer hospital stays and significantly more costly total hospital charges. Increased complications and higher complexity of management in such patients are likely multifactorial in nature. There is a consensus amongst investigators that the treatment of either condition simultaneously results in a series of highly complex decision-making. Cancer-associated thrombosis is one such example that would be difficult to manage especially in patients with concurrent HS. Malignancy is also associated with chronic inflammation, which is known to worsen the progression of aortic stenosis [15].

Outcomes of cancer patients after undergoing transcatheter aortic valve implantation (TAVI) are significantly worse compared to those without cancer. Aortic valve replacement AVR is considered the gold standard of treatment for patients with HS, however, patients with malignancy have been found to have an increased mean pressure gradient even after TAVI, indicating that the risk of HS would persist even after this intervention [16].

Sources of error in this research endeavor stem from the use of the NIS database. While correlations may be made from this data source, the correlations do not equal causation, therefore definitive conclusions cannot be drawn in terms of the increased rates of LGIB and IDA being associated specifically with AS and the formation of colonic AVMs from AvWS. Furthermore, the use of ICD-9 codes to identify diagnosis is also a source of error. Some patients may not have all their known diagnoses identified during a hospitalization, therefore patients with a known history of AS may be underdiagnosed if they were hospitalized with LGIB or IDA, and vice versa. 

Future avenues of research can involve retrospective chart reviews in which CRC patients with known AS are stratified based on AS severity, which can then be correlated with rates of LGIB. If stepwise correlations between AS severity and LGIB severity can be made, then stronger correlations may be deduced between the two conditions. Alternate avenues of research can potentially involve the measurement of vWF levels in patients with AS, then correlating vWF levels to colonoscopy findings (assessing for the presence of AVMs) and severity of LGIB and IDA (measured by hemoglobin and iron levels respectively). In addition, as it is known that aortic valve replacement is curative for Heyde's syndrome and significantly improves postoperative outcomes, comparing pre and post-AVR patients and associated outcomes is a potential area of study for such patients. For all these potential future studies, one must control for patients with CRC and a projected survival of less than one year, as these patients would be unsuitable candidates for TAVI and might therefore have worse mortality from symptomatic severe AS that would be left untreated. 

## Conclusions

Aortic stenosis can be seen as a prognostic risk factor in patients with colorectal cancer for adverse outcomes. As such, the identification of such patients is warranted. Concomitant stratification for the presence of LGIB in such patients would clarify the implication of aortic stenosis in this population. Further investigation into this topic is useful in clinical decisions.
